# Plant Compounds Enhance the Assay Sensitivity for Detection of Active *Bacillus cereus* Toxin

**DOI:** 10.3390/toxins7030835

**Published:** 2015-03-11

**Authors:** Reuven Rasooly, Bradley Hernlem, Xiaohua He, Mendel Friedman

**Affiliations:** 1 Foodborne Contaminants Research Unit, Agricultural Research Service, United States Department of Agriculture, Albany, CA 94710, USA; E-Mails: bradley.hernlem@ars.usda.gov (B.H.); xiaohua.he@ars.usda.gov (X.H.); 2 Healthy Processed Foods Research Unit, Agricultural Research Service, United States Department of Agriculture, Albany, CA 94710, USA; E-Mail: mendel.friedman@ars.usda.gov

**Keywords:** food poisoning, *Bacillus cereus* bacteria, inactivation, *Bacillus cereus* toxin, enterotoxins, plant compounds, plant extracts, cell based assay, food safety

## Abstract

*Bacillus cereus* is an important food pathogen, producing emetic and diarrheal syndromes, the latter mediated by enterotoxins. The ability to sensitively trace and identify this active toxin is important for food safety. This study evaluated a nonradioactive, sensitive, *in vitro* cell-based assay, based on *B. cereus* toxin inhibition of green fluorescent protein (GFP) synthesis in transduced monkey kidney Vero cells, combined with plant extracts or plant compounds that reduce viable count of *B. cereus* in food. The assay exhibited a dose dependent GFP inhibition response with ~25% inhibition at 50 ng/mL toxin evaluated in culture media or soy milk, rice milk or infant formula, products associated with food poisonings outbreak. The plant extracts of green tea or bitter almond and the plant compounds epicatechin or carvacrol were found to amplify the assay response to ~90% inhibition at the 50 ng/mL toxin concentration greatly increasing the sensitivity of this assay. Additional studies showed that the test formulations also inhibited the growth of the *B. cereus* bacteria, likely through cell membrane disruption. The results suggest that the improved highly sensitive assay for the toxin and the rapid inactivation of the pathogen producing the toxin have the potential to enhance food safety.

## 1. Introduction

*Bacillus cereus* a Gram-positive, rod-shaped, beta hemolytic bacterium with an infective dose as low as 10^3^ bacteria per gram of food, is an important cause of foodborne pathogenesis. In the United States up to 84,000 cases of food poisoning occur each year, resulting in major product recalls and substantial economic loss. This bacterium contaminates numerous foods including infant formula [1], infant rice cereal [2], cooked rice [3], dried milk products [4], dehydrated potato products [5], eggs, meat, and spices [6], causing illness worldwide. The heat resistant nature of *Bacillus* spores allows them to survive in foods, which have undergone moderate heat processing and normal cooking processes. The pathogen was first recognized in 1949, after an outbreak of diarrheal food poisoning at a hospital in Oslo, Norway [7]. *B. cereus* produces toxins causing two different types of food poisoning: emetic and diarrheal syndromes [8]. The diarrheal type of food poisoning is caused by enterotoxins produced during vegetative growth of bacteria in the small intestine [9], which act on the epithelial cells, causing massive secretion of fluid into the intestinal lumen, leading to diarrhea [10].

A comprehensive review by Granum [11] notes that: (a) so-called psychrotolerant strains of *B. cereus* are present in dairy and other food products that are heat treated below 100 °C; (b) growth of the bacteria seems to be prevented below pH 4.5; (c) growth of the bacteria is supported in a large variety of foods if not maintained at temperatures below 4 °C or above 60 °C; and (d) 27 different food varieties have been involved in *B. cereus* food poisoning. 

It is not known whether the growth and inhibition of growth of antibiotic-resistant *B. cereus* bacteria that are reported to contaminate food [12,13,14], would be governed by the same temperature and pH parameters observed with the nonresistant (susceptible) bacteria as well as whether toxins produced by resistant pathogenic bacteria differ from those produced by susceptible bacteria. 

The specific objective of the present study was to evaluate a nonradioactive, sensitive, *in vitro* cell-based bioassay for quantitative detection of biologically active *B. cereus* toxins. This bioassay is based on the inhibition of protein synthesis by the *B. cereus* toxin, thus the resulting inhibition of the green fluorescent protein (GFP) intensity in transduced Vero cells, without added substrates or the use of cell fixation methods. As part of this effort, we also determined the effect of plant extracts on the bacterium viable count in foods that were associated with food poisoning outbreaks.

## 2. Results and Discussion

### 2.1. Plant Compounds Reduce the Viable Count of B. cereus in Food

The inhibition of pathogens has been reported by one of the most common and widespread groups of plant secondary metabolites, the polyphenolic compounds, a class of compounds distinguished by their electron rich aromatic moieties and ionizable OH groups. To monitor the effectiveness of plant compounds on *B. cereus* viability in food items that have been involved in *B. cereus* outbreaks, soy milk, infant formula Similac^®^ and control LB media were spiked with *B. cereus* and then treated with the plant formulation of interest and cultured on LB plates. After overnight incubation at 37 °C, there was a 100% reduction of viable counts in the carvacrol (4-isopropyl-2-methyl-phenol) treatment. These plant secondary metabolites that exhibit a strong affinity for proteins and to cell membranes contribute significantly to the bactericidal effect against *B. cereus.* The viability reduction was comparable to the medicinal antibiotic tetracycline. A slight reduction was observed in the epicatechin treatment but significantly different than the PBS control. These results are summarized in Table 1.

**Table 1:** Plant compounds reduce the viable count of *B. cereus* in food.

Treatment	% CFU reduction		
	LB	Similac	Soy milk
green tea extract	89	97	77
green tea extract + carvacrol	100	100	100
green tea extract + bitter almond essential oil	65	77	88
carvacrol	99	99	100
bitter almond essential oil	67	87	93
epicatechin	2	53	45
epigallocatechin gallate	99	84	90
PBS	0	0	0
tetracycline	100	100	100

### 2.2. Detection of B. cereus Toxins

To detect active *B. cereus* toxin in various foods that have been associated with *B. cereus* food poisoning, we developed a bioassay that monitors the inhibition of protein synthesis by *B. cereus* toxins. We generated adenoviral vectors that encode and express the GFP gene (Ad-GFP) under the control of the Cytomegalovirus promoter. In this bioassay, the inhibition of GFP fluorescence intensity in transduced cells was used as a measurement of the biological activity of *B. cereus* toxins. 

**Figure 1:**
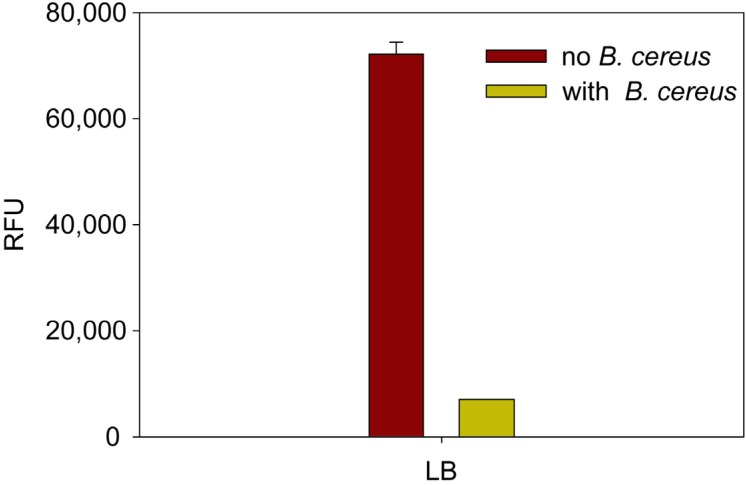
*B. cereus* supernatant decreases GFP expression in transduced African Green monkey epithelial kidney cells. GFP-transduced Vero cells were treated with *B. cereus* supernatants, after incubation for 48 h GFP expression was quantified fluorometrically. Error bars represent standard errors.

*Bacillus cereus* ATCC10876 (NCTC7464) was grown in Luria Broth (LB) at 37 °C for 24 h. The bacteria were harvested by centrifugation and the culture supernatant (15 μL) was used to test for the production of components that inhibit protein synthesis and decrease fluorescence emission in transduced Vero cells. As shown in Figure 1, *B. cereus* toxins inhibited protein synthesis, causing a decrease in GFP expression compared to control. These results suggest that Ad-GFP transduced Vero cell lines can be used for detection of active *B. cereus* toxins.

#### 2.2.1. Dose-Dependent Inhibition of GFP Protein Synthesis in Transduced Vero Cells by *Bacillus cereus* Toxins

**Figure 2:**
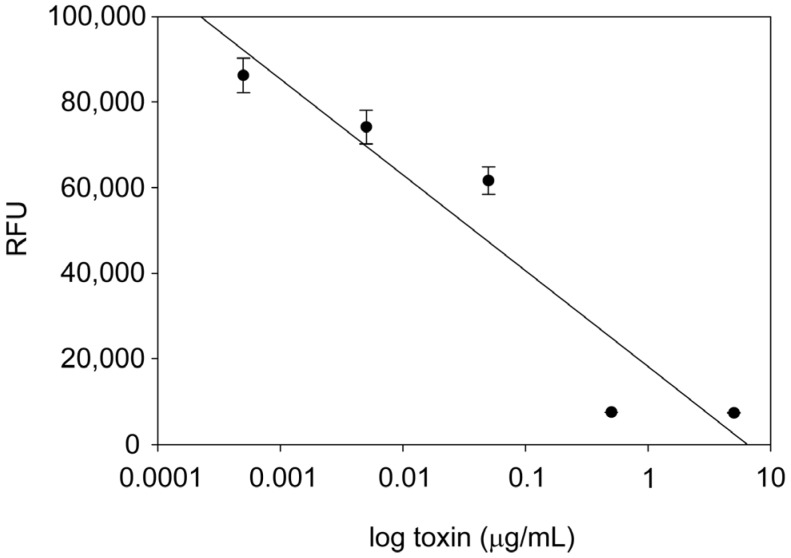
Decrease in green fluorescent protein (GFP) expression in transduced Vero cells with Ad-CMV-GFP in the presence of increasing concentrations of *B. cereus* toxin. GFP expression was quantified fluorometrically. Error bars represent standard errors (*n* = 3).

To verify that *B. cereus* toxins complex can inhibit protein synthesis in the same manner as *B. cereus* culture supernatant, increasing concentrations of *B. cereus* toxins ranging from 5 ng/mL to 500 ng/mL were added to transduced Vero cells. The fluorometrically quantified result in Figure 2, indicates that the GFP fluorescence emission decreased in a dose-dependent manner. A further decrease of *B. cereus* toxins concentration to below 5 ng/mL abolished inhibition of protein synthesis and the relative fluorescence returned to the background level.

#### 2.2.2. Detection of Active *B. Cereus* Toxins in Different Food Items

**Figure 3:**
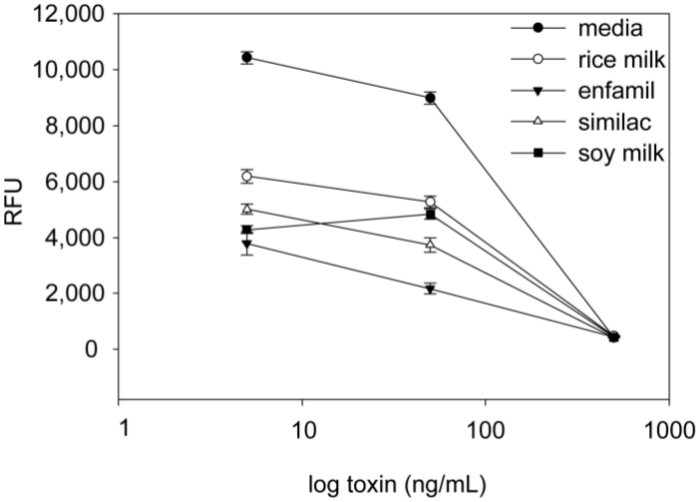
Detection of active *B. cereus* toxin in soy milk, Similac^®^, Enfamil^®^, rice milk and media. The food components were spiked with *B. cereus* toxin at concentration of 500 ng/mL, 50 ng/mL and 5 ng/mL. Fifteen microliters of spiked milk with 85 μL of media were incubated for 48 h in transduced Vero cells. GFP expression was quantified fluorometrically, with the plot showing relative fluorescence units (RFU). Error bars represent standard errors (*n* = 3).

We evaluated the ability of the assay to detect active *B. cereus* toxins in different food items that were associated with *B. cereus* food poisoning, specifically commercial rice milk, soy milk and the liquid infant formulae Similac^®^ and Enfamil^®^ (Mead Johnson, Glenview, IL, USA). The liquid foods and control media were spiked with increasing concentration of *B. cereus* toxins (5, 50, 500 ng). These spiked food items were diluted into media and added to the transduced Vero cells. After incubation for 48 h, GFP expression was measured fluorometrically. As shown in Figure 3, food matrix effects seem to increase the signal-to-noise ratio and reduce GFP expression. However, the GFP expression in food with *B. cereus* toxins was statistically significantly lower from the same food item spiked with low concentration (5 ng/mL) of *B. cereus* toxins (*p* < 0.05). These results suggest that this diagnostic method can be used for detection of *B. cereus* toxin in a variety of food products.

**Figure 4:**
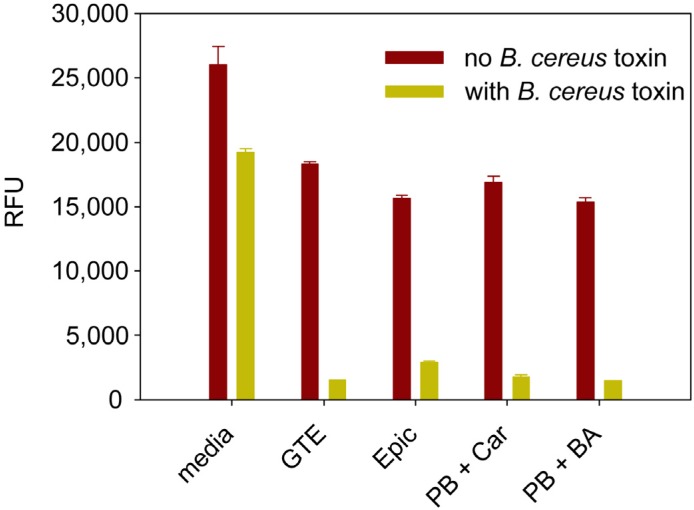
Enhancement of the biological activities of 50 ng/mL of *B*. *cereus* toxin by green tea extracts (GTE), epicatechin (Epic), carvacrol (Car), and bitter almond essential oil (BA).

#### 2.2.3. Enhancement of *B. cereus* Toxins Detection Using Natural Tea Compounds

We previously reported that apple juice inhibited Shiga toxin 2 (Stx2) [15,16]. It was therefore of interest to investigate whether plant compounds would also inhibit *B. cereus* growth and the biological activity of *B. cereus* toxins. Our results (Table 1) show that plant extracts such as bitter almond essential oil or green tea extract that contain high quantities of carvacrol, epicatechin, and epicatechin gallate suppress *B. cereus* growth in LB agar-plates, rice milk, infant formula, and soy milk. We added the above plant extracts to *B. cereus* toxins to determine if they can inhibit *B. cereus* toxins activity. As shown in Figure 4, to our surprise, we found that the plant compounds and extracts enhanced the biological activity of *B. cereus* toxins and increased the assay sensitivity.

### 2.3. Discussion

The main objective of this study was to replace the rabbit ileal loop assay which is the current bioassay for detection of biologically active *Bacillus* diarrhoeal enterotoxin, the toxin which has been associated with food poisoning outbreaks caused by this organism. This *in vivo* assay is based on the fact that the toxin elicits diarrhea by disrupting the integrity of the plasma membrane of epithelial cells in the small intestine. This procedure involves inoculation of supernatant from suspected culture into a ligated segment of ileum, which responds by secretion of fluids into the loop of intestine [17]. However, this *in vivo* methodology raises ethical concerns with regard to the use of experimental animals. Sensitive, rapid, and specific methods such as the polymerase chain reaction (PCR) assays have been used [18,19], but these indirect methods measure *B. cereus* genes and thus do not detect toxin gene expression. Also, the PCR-based assays do not provide a direct measurement of the toxin level and do not distinguish between live and dead organisms and active and inactive toxins. The immunoassay methods that have been used cannot replace the rabbit ileal loop assay because the immunoassay kits that are available can detect the presence of only NheA, one of the three toxin components [20], even though the other two components, NheB and NheC, are essential for binding to cell membranes and all three components are necessary to maximize toxic activity. Furthermore, the immunoassays also do not distinguish between active and inactive toxins, a key requirement when studying inhibition of toxins by plant compounds. 

An alternative cell bioassay measures the uptake of radiolabeled C^14^-leucine across the epithelial plasma membrane and its incorporation into Vero cells [21]. Inhibition of protein synthesis by the toxin leads to reduced uptake of the radiolabeled leucine. 

To improve upon the radiolabel assay and avoid the use of radioactive materials, in the present study we generated and used adenoviral vectors that encode and express the GFP gene (Ad-GFP). For quantitative detection of the biologically active *Bacillus* toxin we fluorometrically measured the inhibition of the development of GFP fluorescence intensity in transduced Vero cells. 

The results of the present study show that when plant extracts and plant polyphenolic compounds were added to food items containing *B. cereus* they reduced the viable bacterium counts. The results of our previous study showed that when apple juice, which is naturally rich in polyphenolic compounds, was added to toxin, it significantly reduced the biological activity of staphylococcal enterotoxin A [15,16]. It was therefore of interest to investigate whether these antimicrobial plant extracts and plant polyphenolic compounds would also inhibit the biological activity of the *B. cereus* toxin.

Our data in Figure 4 show that these plant extracts alone had a minor effect on Vero cell expression of GFP, compared with the same extracts combined with *B. cereus* toxin. However, rather than inhibit the toxin we observed an enhancement of the biological activity of the toxin and amplification of the sensitivity of the assay. This observation may be important in timing the additions of certain antimicrobials to foods, because addition to foods containing *B. cereus* that has already formed toxin could lead to increased toxicity.

We do not know the mechanism that governs the observed enhancement in the bioactivity of the toxin. One possibility is that the plant compounds act synergistically with toxin at the receptor sites of the cell, the plant lipophilic-hydrophilic (amphiphilic) compounds that possess a high affinity for cell membranes bind the toxin through their hydroxyl groups and may enhance accumulation in the cell membrane. Another possibility is that the plant compounds stress the Vero cells by binding or interacting with the cell membrane [22,23], resulting in an enhanced susceptibility to the toxin. A third possibility is that the antioxidant or hydrogen-binding properties of the phenolic OH groups [24] enhance cytotoxicity by altering the structures (conformations) or ionic properties (isoelectric point) of the protein toxin or the Vero cell receptor sites. These aspects merit further study. 

Unlike carvacrol, the tea catechins are completely water-soluble, and so it is unlikely that the hydrophobic parts of the molecules affect bioactivity of the toxin. Carvacrol with numerous health-promoting properties is a major component of plant essential oils such as oregano oil used as a salad dressing [25]. Because the results of the present study show that carvacrol seems to be the most effective compound in enhancing the assay sensitivity of the *Bacillus* *cereus* toxin, it also has relevance for the safety of this foodborne enterotoxin.

## 3. Experimental Section

### 3.1. Materials

Liquid foods were obtained from a local store; the *Bacillus cereus* toxin was a gift from Toxin Technology (Sarasota, FL, USA); carvacrol, epicatechin, epicatechin gallate were obtained from Sigma (St. Louis, MO, USA), and bitter almond essential oil (bitter almond EO) was obtained from Lhasa Karnak Herb Co. (Berkeley, CA, USA). Human Embryonic Kidney 293 cells (HEK293) (ATCC CRL-1573), *Bacillus cereus* ATCC10876 and Vero African Green Monkey adult kidney cells (ATCC CCL-81) were obtained from the American Type Culture Collection (Manassas, VA, USA). Green tea extract was prepared as previously described [26], by extracting green tea leaves into boiling 10% EtOH in water, and freeze drying the filtrate.

### 3.2. Test Substances

The seven test substances were prepared as follows:

1. Green tea extract (0.02%)—2 mg + 100 μL EtOH; add 3.9 mL PBS pH 7.0; vortex 1 min; add 6 mL PBS pH 7.0; clear, very slight greenish tinge.
2. GTE (0.02%) + carvacrol (Sigma, St. Louis, MO, USA) (0.1%)—3 μL carvacrol + 30 μL EtOH; add 2.967 mL GTE (0.02%) pH 7.0; vortex 1 min; clear, very slight greenish tinge.
3. GTE (0.02%) + bitter almond essential oil (0.1%) (Lhasa Karnak)—3 μL bitter almond EO + 30 μL EtOH; add 2.967 mL GTE (0.02%) pH 7.0; vortex 1 min; clear, very slight greenish tinge.
4. Carvacrol (0.1%)—3 μL carvacrol + 30 μL EtOH; add 2.967 mL PBS pH 7.0; vortex 1 min; very slightly yellow.
5. Bitter almond essential oil (0.1%)—3 μL bitter almond EO + 30 μL EtOH; add 2.967 mL PBS pH 7.0; vortex 1 min; clear.
6. Epicatechin (Sigma) (0.02%)—2 mgs in 100 μL EtOH; add 3.9 mL PBS pH 7.0; vortex 1 min; add 6 mL PBS pH 7.0; clear solution.
7. Epigallocatechin gallate (Chromadex, Irvine, CA, USA) (0.02%)—2 mgs in 100 μL EtOH; add 3.9 mL PBS pH 7.0; vortex 1 min; add 6 mL PBS pH 7.0, clear.

All formulations were adjusted to pH 7.0 for comparative evaluation of efficacy. Ethanol was added to keep active compounds in solution.

### 3.3. Determination of Bactericidal Effect of Plant Compound by Viable Bacteria Cell Counts

The effects of plant compounds on *B. cereus* bacterial growth in Soy milk and Similac^®^ (Abbott, Abbott Park, IL, USA) milk formula were studied by adding 90 µL of various plant compounds to the mid-logarithmic growth phase 10 µL of milk containing 100 colony-forming units (CFU), then plated on Luria Broth (LB) agar plates. The CFU were counted following incubation for 24 h at 37 °C. 

### 3.4. Determination of Toxin Activity

#### 3.4.1. Cell Culture

Vero cell and HEK293 cells were maintained in DMEM (Dulbecco’s Modified Eagle Medium, Life Technologies, Grand Island, NY, USA) containing 10% fetal bovine serum (FBS) and 100 units/mL of both penicillin and streptomycin. Cells were trypsinized before harvesting.

#### 3.4.2. Generation of Adenoviral Vectors that Express the GFP Gene

To visualize the effect of active *Bacillus* toxin on living cells, we measured changes in GFP expression levels. The GFP gene was isolated from the Green Lantern vector (BRL) by digestion with the *Not I* restriction enzyme. The 750 bp fragment was purified from the gel using a Qiagen kit (Duesseldorf, Germany) and was subcloned into the *Not I* site of the adenoviral shuttle plasmid between the Cytomegalovirus immediate-early promoter (CMV) and the polyadenylation signal from bovine growth hormone. The plasmid pJM17 containing the full length of the adenovirus genome including a 4.4 Kb sequence of antibiotics resistance gene were co-transfected in HEK293 cells with the shuttle plasmid containing the GFP gene flanked by the adenovirus E1 sequences. After 10 days, the cytopathic effect appeared and the transfected cells became round and detached from the plate. The cells were then analyzed by fluorescence microscopy to detect GFP gene expression. Individual plaques of Ad-GFP were amplified. 

#### 3.4.3. Plaque Assays for Purification and Titration of the Adenovirus

Plaque assays depend on the ability of the adenovirus to propagate in HEK293 cells. Six 35 mm tissue culture plates were seeded with HEK293 cells. The cells were incubated at 37 °C in a 5% CO_2_ incubator until they were 90% confluent. Serial dilutions were made in DMEM in the medium supplemented with 2% FBS. The diluted virus was added to the cells. After 2 h, the medium was removed and replaced with 1× Modified Eagle's Medium (MEM) and 1% agarose (SeaPlaque, Lonza Ltd., Basel, Switzerland). The agar overlay was added to keep the virus localized after the cells had lysed. After 5 days, plaques were visible, and were counted for titer determination after 7 days.

#### 3.4.4. Quantifying *B. cereus* Toxin Activity

Vero cells were plated on black 96-well plates (Greiner 655090 obtained from Sigma) at 1 × 10^4^ cells in 100 µL of medium per well. Cells were incubated overnight to allow time for cells to attach to the plate. Treated samples were then added to each well and incubated for 48 h at 37 °C in a 5% CO_2_ incubator. The cells were then transduced with Ad-GFP at a Multiplicity of Infection (MOI) of 100 for 48 h (100 Plaque Forming Units (pfu) per cell). The medium was removed, and cells were washed three times with pH 7.4 phosphate buffered saline (PBS). Quantification of fluorescence emission by the cells expressing GFP was measured using a 528/20 nm emission filter and 485/20 nm excitation filter in a Synergy HT Multi-Detection Microplate Reader (BioTek, Winooski, VT, USA).

### 3.5. Statistical Analysis

Statistical analysis was performed with SigmaStat 3.5 for Windows (Systat Software, San Jose, CA, USA). Multiple comparisons among transduced treated cells were made. One-way analysis of variance (ANOVA) was used to compare transduced treated cells to transduced untreated cells. The experiments were repeated at least three times, and results with *p* < 0.05 were considered statistically significant. 

## 4. Conclusions

The safety of contaminated food depends on our ability to identify low levels of the active toxin. In the present study, we first determined the levels of the *Bacillus cereus* toxin in commercial soy milk, rice milk, and baby formula using a nonradioactive *in vitro* cell assay based on toxin inhibition of a green fluorescent protein (GFP) synthesis in transduced monkey kidney Vero cells. In the course of the experimental studies, we discovered that added plant compounds significantly amplified the assay response to ~90% inhibition at the 50 ng/mL toxin concentration. The results suggest that the large increase in the sensitivity of the assay for the virulent toxin can contribute to the enhancement of food safety. Further studies are needed to determine the mechanism of the amplification of the assay response and the application of the highly sensitive assay to the *Bacillus cereus* toxin in different environments, including commercial foods and animal and human tissues.
